# Diethyl 2,2′-(1,4-phenyl­enedi­oxy)­diacetate

**DOI:** 10.1107/S1600536812030747

**Published:** 2012-07-10

**Authors:** Jérôme Husson, Michael Knorr, Yoann Rousselin, Marek M. Kubicki

**Affiliations:** a Institute UTINAM UMR CNRS 6213, University of Franche-Comté, 16 Route de Gray, Besançon 25030, France; b ICMUB UMR CNRS 5260, University of Bourgogne, 9 Avenue A. Savary, Dijon 21078, France

## Abstract

In the title compound, C_14_H_18_O_6_, a crystallographic center at the centroid of the aromatic ring generates the complete mol­ecule which is planar within 0.085 (1) Å for the non-H atoms. In the crystal, weak C—H⋯O and C—H⋯π inter­actions link the molecules.

## Related literature

For the syntheses and applications of aryl­oxyacetic acid derivatives, see: Carter & Lawrence (1900 ▶); Moser (1950 ▶); Kassem (1997 ▶); Hodge *et al.* (2000 ▶). For related crystal structures, see: Zhuang & Wang (2009 ▶); Du *et al.* (2006 ▶); Gao *et al.* (2004 ▶).
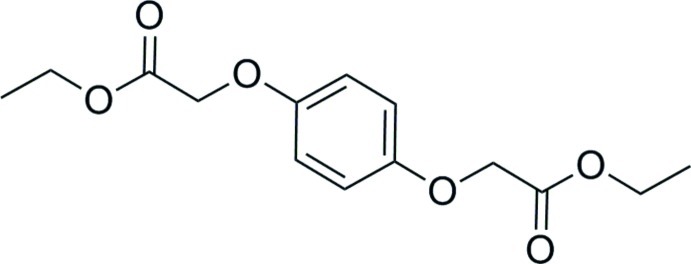


## Experimental

### 

#### Crystal data

C_14_H_18_O_6_
*M* *_r_* = 282.28
Monoclinic, 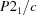

*a* = 4.9254 (3) Å
*b* = 9.7194 (5) Å
*c* = 14.9170 (11) Å
β = 108.313 (3)°
*V* = 677.94 (7) Å^3^
*Z* = 2
Mo *K*α radiation
μ = 0.11 mm^−1^
*T* = 115 K
0.40 × 0.27 × 0.15 mm

#### Data collection

Nonius KappaCCD diffractometer
2560 measured reflections
1536 independent reflections
1344 reflections with *I* > 2σ(*I*)
*R*_int_ = 0.029

#### Refinement

*R*[*F* ^2^ > 2σ(*F* ^2^)] = 0.045
*wR*(*F* ^2^) = 0.114
*S* = 1.09
1536 reflections
92 parameters
H-atom parameters constrained
Δρ_max_ = 0.29 e Å^−3^
Δρ_min_ = −0.26 e Å^−3^

### 

Data collection: *COLLECT* (Nonius, 2004 ▶); cell refinement: *SCALEPACK* (Otwinowski & Minor, 1997 ▶); data reduction: *DENZO* (Otwinowski & Minor, 1997 ▶) and *SCALEPACK*; program(s) used to solve structure: *SIR92* (Altomare *et al.*, 1993 ▶); program(s) used to refine structure: *SHELXL97* (Sheldrick, 2008 ▶); molecular graphics: *ORTEP-3* (Farrugia, 1997 ▶); software used to prepare material for publication: *WinGX* (Farrugia, 1999 ▶).

## Supplementary Material

Crystal structure: contains datablock(s) I, global. DOI: 10.1107/S1600536812030747/jj2131sup1.cif

Structure factors: contains datablock(s) I. DOI: 10.1107/S1600536812030747/jj2131Isup3.hkl

Supplementary material file. DOI: 10.1107/S1600536812030747/jj2131Isup4.cdx

Supplementary material file. DOI: 10.1107/S1600536812030747/jj2131Isup4.cml

Additional supplementary materials:  crystallographic information; 3D view; checkCIF report

## Figures and Tables

**Table 1:** Hydrogen-bond geometry (Å, °) *Cg* is the centroid of the C1–C3/C1^i^–C3^i^ ring.

*D*—H⋯*A*	*D*—H	H⋯*A*	*D*⋯*A*	*D*—H⋯*A*
C4—H4*B*⋯*Cg* ^i^	0.99	2.57	3.426 (2)	145
C6—H6*B*⋯O1^ii^	0.99	2.65	3.340 (2)	127
C6—H6*B*⋯O2^ii^	0.99	2.68	3.401 (2)	130

Symmetry codes: (i) 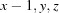; (ii) 
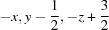
.

**Fig. 1.**
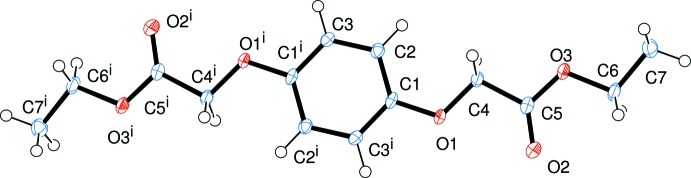
Molecular structure of title compound(I) showing the atom labeling scheme of the asymmetric unit and 50% probability displacement ellipsoids. A crystallographic inversion center at the centroid of the central aromatic ring generates the complete molecule.

**Fig. 2.**
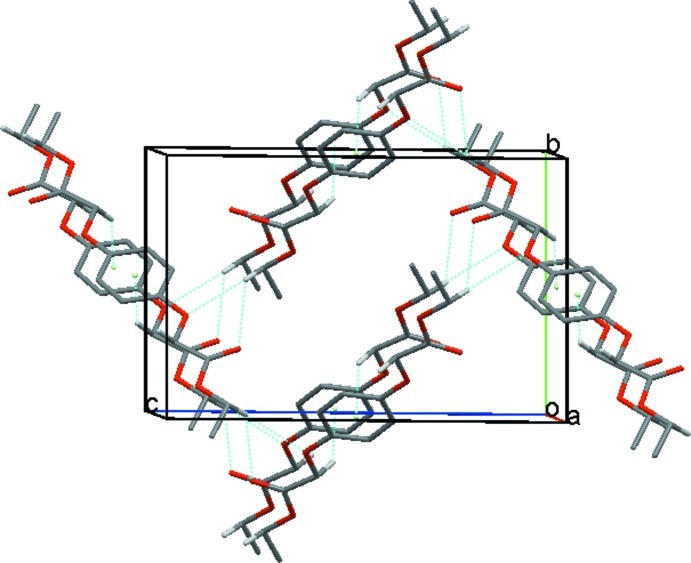
Packing diagram of the title compound viewed roughly along the a axis. Dashed lines indicate weak C—H···π (represented by the centroid of aromatic ring) and C—H···O intermoleclar interactions. Hydrogen atoms not involved in these interactions have been removed for clarity.

## supplementary crystallographic information

### Comment

The title compound has been synthesized by different paths including the reaction of hydroquinone with sodium ethoxide followed by a Williamson reaction of the resulting dianion with ethyl bromoacetate (Carter & Lawrence, 1900), or the esterification of the corresponding diacid in the presence of BF_3_—Et_2_O complex as a catalyst (Moser, 1950). It has been used in the preparation of polymers (Kassem, 1997) and polyrotaxanes (Hodge *et al.*, 2000).

A crystallographic center at the centroid of the central aromatic ring generates the complete molecule which is planar within 0.085 (1)Å without the H atoms (Fig. 1). The largest deviation from planarity among the ten non-hydrogen atoms is derived from O1 (0.085 (1) Å). A similar molecular geometry has been reported for the analogous dimethyl 1,4-(*p*-phenylenedioxy)diacetate molecule (Zhuang & Wang, 2009) as well as for the corresponding diacid (Du *et al.*, 2006) and dianion (Gao *et al.*, 2004). Weak intermolecular C—H···O and C—H··· π-ring (methylene···aryl) interactions (Table 1, Cg is the centroid of the C1-C3/C1i-C3i π-ring) are observed which contribute to crystal packing in the crystal (Fig. 2).

### Experimental

Sodium (4.60 g; 0.2 mol) was added portionwise to absolute ethanol (250 ml). Once all sodium reacted, hydroquinone (11.00 g; 0.1 mol) was added and the solution refluxed for five minutes. After cooling to room temperature, ethyl chloroacetate (21.3 ml; 0.1 mol) was added and the reaction mixture refluxed for five hours. The mixture was then poured onto distilled water (250 ml) and pH adjusted to 3 by addition of few drops of concentrated hydrochloric acid. The aqueous layer was extracted with methyl-tertbutyl ether (4τimes100 ml). The organic layers were then combined, washed with saturated sodium hydrogencarbonate (3τimes100 ml) and water (100 ml). The ethereal layer was dried over calcium sulfate and concentrated under vacuum to afford the title compound as a beige solid. The crude product was recrystallized from dilute ethanol to afford the pure compound as colorless needles (5.58 g, 47%).

### Refinement

All H atoms were placed in calculated positions and treated in a riding model. C–H distances were set to 0.95 Å (aromatic), 0.99 Å (methylene) and 0.98 Å (methyl) with *U_iso_*(H) = *xU_eq_*(C), where *x* = 1.5 for methyl and 1.2 for aromatic and methylene H atoms.

### Crystal data

**Table:**

C_14_H_18_O_6_	*F*(000) = 300
*M**_r_* = 282.28	*D*_x_ = 1.383 Mg m^−^^3^
Monoclinic, *P*2_1_/*c*	Mo *K*α radiation, λ = 0.71073 Å
Hall symbol: -P 2ybc	Cell parameters from 1387 reflections
*a* = 4.9254 (3) Å	θ = 1.0–27.5°
*b* = 9.7194 (5) Å	µ = 0.11 mm^−^^1^
*c* = 14.9170 (11) Å	*T* = 115 K
β = 108.313 (3)°	Prism, colourless
*V* = 677.94 (7) Å^3^	0.40 × 0.27 × 0.15 mm
*Z* = 2	

### Data collection

**Table:**

Nonius KappaCCD diffractometer	1344 reflections with *I* > 2σ(*I*)
Radiation source: Enraf–Nonius FR590	*R*_int_ = 0.029
Horizonally mounted graphite crystal monochromator	θ_max_ = 27.4°, θ_min_ = 2.5°
Detector resolution: 9 pixels mm^-1^	*h* = −6→6
CCD rotation images, thick slices scans	*k* = −9→12
2560 measured reflections	*l* = −19→19
1536 independent reflections	

### Refinement

**Table:**

Refinement on *F*^2^	Primary atom site location: structure-invariant direct methods
Least-squares matrix: full	Secondary atom site location: difference Fourier map
*R*[*F*^2^ > 2σ(*F*^2^)] = 0.045	Hydrogen site location: inferred from neighbouring sites
*wR*(*F*^2^) = 0.114	H-atom parameters constrained
*S* = 1.09	*w* = 1/[σ^2^(*F*_o_^2^) + (0.0388*P*)^2^ + 0.4496*P*] where *P* = (*F*_o_^2^ + 2*F*_c_^2^)/3
1536 reflections	(Δ/σ)_max_ < 0.001
92 parameters	Δρ_max_ = 0.29 e Å^−^^3^
0 restraints	Δρ_min_ = −0.26 e Å^−^^3^
0 constraints	

### Special details

**Table:**

Geometry. All e.s.d.'s (except the e.s.d. in the dihedral angle between two l.s. planes) are estimated using the full covariance matrix. The cell e.s.d.'s are taken into account individually in the estimation of e.s.d.'s in distances, angles and torsion angles; correlations between e.s.d.'s in cell parameters are only used when they are defined by crystal symmetry. An approximate (isotropic) treatment of cell e.s.d.'s is used for estimating e.s.d.'s involving l.s. planes.
Refinement. Refinement of *F*^2^ against ALL reflections. The weighted *R*-factor *wR* and goodness of fit *S* are based on *F*^2^, conventional *R*-factors *R* are based on *F*, with *F* set to zero for negative *F*^2^. The threshold expression of *F*^2^ > σ(*F*^2^) is used only for calculating *R*-factors(gt) *etc*. and is not relevant to the choice of reflections for refinement. *R*-factors based on *F*^2^ are statistically about twice as large as those based on *F*, and *R*- factors based on ALL data will be even larger.

### Fractional atomic coordinates and isotropic or equivalent isotropic displacement parameters (Å^2^)

**Table:**

	*x*	*y*	*z*	*U*_iso_*/*U*_eq_	
O1	0.2542 (2)	0.85579 (11)	0.61485 (7)	0.0183 (3)	
O2	0.0149 (3)	0.73769 (12)	0.73286 (7)	0.0253 (3)	
O3	−0.2554 (2)	0.60124 (11)	0.61710 (7)	0.0204 (3)	
C1	0.3679 (3)	0.92498 (14)	0.55420 (10)	0.0153 (3)	
C2	0.2808 (3)	0.90691 (14)	0.45650 (10)	0.0160 (3)	
H2	0.1321	0.8440	0.4268	0.019*	
C3	0.4152 (3)	0.98254 (15)	0.40315 (10)	0.0162 (3)	
H3	0.3575	0.9706	0.3366	0.019*	
C4	0.0340 (3)	0.76022 (14)	0.57334 (10)	0.0164 (3)	
H4A	0.1066	0.6860	0.5415	0.020*	
H4B	−0.1269	0.8066	0.5259	0.020*	
C5	−0.0652 (3)	0.70087 (15)	0.65171 (10)	0.0174 (3)	
C6	−0.3690 (3)	0.53564 (16)	0.68587 (10)	0.0207 (3)	
H6A	−0.4725	0.6039	0.7122	0.025*	
H6B	−0.2112	0.4965	0.7384	0.025*	
C7	−0.5696 (4)	0.42320 (16)	0.63551 (11)	0.0235 (3)	
H7A	−0.7226	0.4628	0.5829	0.035*	
H7B	−0.6530	0.3786	0.6797	0.035*	
H7C	−0.4639	0.3550	0.6112	0.035*	

### Atomic displacement parameters (Å^2^)

**Table:**

	*U*^11^	*U*^22^	*U*^33^	*U*^12^	*U*^13^	*U*^23^
O1	0.0220 (5)	0.0212 (5)	0.0149 (5)	−0.0062 (4)	0.0106 (4)	0.0001 (4)
O2	0.0311 (6)	0.0298 (6)	0.0172 (6)	−0.0089 (5)	0.0106 (5)	0.0001 (5)
O3	0.0268 (6)	0.0205 (5)	0.0179 (5)	−0.0062 (4)	0.0128 (4)	0.0005 (4)
C1	0.0176 (7)	0.0154 (6)	0.0170 (7)	0.0024 (5)	0.0114 (5)	0.0030 (5)
C2	0.0177 (7)	0.0154 (6)	0.0175 (7)	0.0001 (5)	0.0091 (5)	−0.0011 (5)
C3	0.0182 (7)	0.0183 (7)	0.0141 (6)	0.0007 (5)	0.0080 (5)	−0.0008 (5)
C4	0.0198 (7)	0.0157 (6)	0.0167 (7)	−0.0023 (5)	0.0099 (5)	0.0001 (5)
C5	0.0187 (7)	0.0169 (7)	0.0193 (7)	0.0013 (5)	0.0100 (5)	0.0031 (5)
C6	0.0254 (8)	0.0218 (7)	0.0190 (7)	−0.0027 (6)	0.0130 (6)	0.0041 (6)
C7	0.0268 (8)	0.0194 (7)	0.0263 (8)	−0.0036 (6)	0.0111 (6)	0.0008 (6)

### Geometric parameters (Å, º)

**Table:**

O1—C1	1.3796 (16)	C3—H3	0.9500
O1—C4	1.4145 (17)	C4—C5	1.5157 (19)
O2—C5	1.2037 (18)	C4—H4A	0.9900
O3—C5	1.3334 (18)	C4—H4B	0.9900
O3—C6	1.4603 (16)	C6—C7	1.506 (2)
C1—C3^i^	1.388 (2)	C6—H6A	0.9900
C1—C2	1.395 (2)	C6—H6B	0.9900
C2—C3	1.3939 (19)	C7—H7A	0.9800
C2—H2	0.9500	C7—H7B	0.9800
C3—C1^i^	1.388 (2)	C7—H7C	0.9800
			
C1—O1—C4	116.52 (11)	H4A—C4—H4B	108.5
C5—O3—C6	115.01 (11)	O2—C5—O3	125.01 (13)
O1—C1—C3^i^	115.31 (12)	O2—C5—C4	125.40 (13)
O1—C1—C2	124.67 (13)	O3—C5—C4	109.59 (12)
C3^i^—C1—C2	120.02 (13)	O3—C6—C7	107.61 (12)
C3—C2—C1	118.99 (13)	O3—C6—H6A	110.2
C3—C2—H2	120.5	C7—C6—H6A	110.2
C1—C2—H2	120.5	O3—C6—H6B	110.2
C1^i^—C3—C2	120.99 (13)	C7—C6—H6B	110.2
C1^i^—C3—H3	119.5	H6A—C6—H6B	108.5
C2—C3—H3	119.5	C6—C7—H7A	109.5
O1—C4—C5	107.51 (11)	C6—C7—H7B	109.5
O1—C4—H4A	110.2	H7A—C7—H7B	109.5
C5—C4—H4A	110.2	C6—C7—H7C	109.5
O1—C4—H4B	110.2	H7A—C7—H7C	109.5
C5—C4—H4B	110.2	H7B—C7—H7C	109.5
			
C4—O1—C1—C3^i^	−179.61 (12)	C6—O3—C5—O2	0.2 (2)
C4—O1—C1—C2	0.03 (19)	C6—O3—C5—C4	−179.79 (11)
O1—C1—C2—C3	−179.44 (13)	O1—C4—C5—O2	5.5 (2)
C3^i^—C1—C2—C3	0.2 (2)	O1—C4—C5—O3	−174.54 (11)
C1—C2—C3—C1^i^	−0.2 (2)	C5—O3—C6—C7	−177.83 (12)
C1—O1—C4—C5	−178.01 (11)		

Symmetry code: (i) −*x*+1, −*y*+2, −*z*+1.

### Hydrogen-bond geometry (Å, º)

Cg is the centroid of the C1-C3/C1i-C3i ring.

**Table:**

*D*—H···*A*	*D*—H	H···*A*	*D*···*A*	*D*—H···*A*
C4—H4*B*···*Cg*^ii^	0.99	2.57	3.426 (2)	145
C6—H6*B*···O1^iii^	0.99	2.65	3.340 (2)	127
C6—H6*B*···O2^iii^	0.99	2.68	3.401 (2)	130

Symmetry codes: (ii) *x*−1, *y*, *z*; (iii) −*x*, *y*−1/2, −*z*+3/2.
